# Cholera in Mozambique, Variant of *Vibrio cholerae*

**DOI:** 10.3201/eid1011.040682

**Published:** 2004-11

**Authors:** M. Ansaruzzaman, N.A. Bhuiyan, G. Balakrish Nair, David A. Sack, Marcelino Lucas, Jacqueline L. Deen, Julia Ampuero, Claire-Lise Chaignat, The Mozambique Cholera Vaccine Demonstration Project Coordination Group

**Affiliations:** * Centre for Health and Population Research, Dhaka, Bangladesh;; † Ministério da Saude, Maputo, Mozambique;; ‡ International Vaccine Institute, Seoul, Korea;; § Médecins Sans Frontières, Geneva, Switzerland;; ¶ Epicentre, Paris, France;; # World Health Organization, Geneva, Switzerland

**Keywords:** letter, Vibrio cholerae, CTX prophage, cholera, classical biotype, El Tor biotype

**To the Editor:** Cholera outbreaks caused by toxigenic *Vibrio cholerae* serogroup O1 frequently occur in many sub-Saharan African countries. The serogroup O1 is classified into two biotypes, classical and El Tor. The seventh and current pandemic of cholera is caused by the El Tor biotype; the classical biotype is believed to be extinct. The classical and El Tor biotypes of *V. cholerae* O1 are closely related in their O-antigen biosynthetic genes but differ in other regions of the genome. The genomic structure of the CTXΦ filamentous phage (*1*), in which the cholera toxin genes are contained, differs between the classical and El Tor biotypes. CTX^class^Φ is found in classical strains, CTX^ET^Φ is present in El Tor and O139 strains, and CTX^calc^Φ is found in resurgent O139 strains. The diversity of CTXΦ among biotypes is mainly due to the variations in the repeat sequence elements, particularly in the *rstR* gene region (*2*).

While conducting surveillance in the cholera treatment center in Beira, the second largest city in Mozambique, we examined 175 rectal swabs or stool samples from January 7 to March 8, 2004, using standard published procedures. During this period, we isolated 58 strains of *V. cholerae* O1. The isolates were transported to the Enteric Microbiology Unit of the International Center for Diarrhoeal Disease Research in Dhaka, Bangladesh (ICDDRD,B), for further phenotypic and genotypic characterization to determine serotype, biotype, and presence of important virulence genes. All 58 strains were identified as *V. cholerae* O1 of the Ogawa serotype. Forty strains selected for detailed characterization were resistant to polymyxin B, agglutinated chicken cells, yielded a positive Voges-Proskauer reaction, were positive for the El Tor hemolysin by the tube agglutination method, and were sensitive to group IV El Tor phage but resistant to the classical group V phage and were therefore classified as the El Tor biotype. The antimicrobial susceptibility of 15 of the 40 isolates examined showed that the strains were sensitive to tetracycline, ampicillin, furazolidone, erythromycin, and ciprofloxacin but resistant to trimethoprim-sulfamethoxazole, and also to the vibriostatic compound 0/129.

By using polymerase chain reaction (PCR), we established that all 40 strains carried the *ctxA* gene (constituent gene of the CTX prophage) and the *tcpA* gene (the El Tor type), a constituent gene of the vibrio pathogenicity island. We then focused on the *rstR* gene because of its diversity between the two biotypes. All of the 40 El Tor strains produced a 500-bp PCR product of the *rstR* gene of the classical type (*rstR*^class^), despite belonging to the El Tor biotype. Nucleotide sequence analysis of the *rstR* gene of two representative Mozambique strains showed 100% homology to the classical *rstR* gene of classical reference strain O395. The amino acid sequence of the B-subunit of classical and El Tor biotypes have distinct signature sequences (*3*). We amplified the *ctxB* gene using specific primers and found that the deduced amino acid sequence of the CT-B subunit of the Mozambique strains varied from the El Tor CT-B subunit at positions 39 (histidine replaces tyrosine in El Tor) and 68 (threonine replaces isoleucine in El Tor), and the amino acid residues at these positions are identical to those of the classical CT-B subunit (Figure). The nucleotide sequences obtained for *ctxB* of the two Mozambique strains B33 and B65 were deposited in GenBank under accession no. AY648939 and AY6448940, respectively. Therefore, the Mozambique strains of *V. cholerae* O1 displayed typical traits of the El Tor biotype overall but carried the classical CTX prophage.

**Figure:**
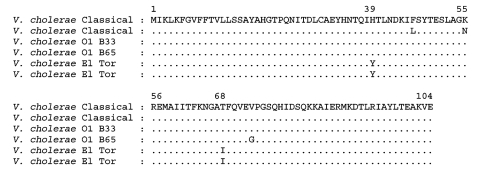
Amino acid sequence alignment of CT-B subunit of *Vibrio cholerae* O1 classical, El Tor, and Mozambique (B33 and B65) strains. Identical amino acid residues are indicated by a period. Amino acid sequences of CtxB of *V. cholerae* classical (AAL60524.1; AAM47189.1) and El Tor (AAM74192.1; AAM77066.1) are from GenBank.

Our findings that El Tor strains of *V. cholerae* O1 from Mozambique are carrying the classical prophage shows the presence of genetic materials associated with the classical biotype in Mozambique. Further, these findings provide the first circumstantial evidence of transmission of the classical CTX prophage. The CTX prophages in El Tor strains give rise to infectious phage particles (1), but neither of the two CTX prophages integrated at two different sites of the classical genome give rise to phage particles (*4*). Subsequent studies have shown that, although the genes of the classical prophages encode functional forms of all of the proteins needed for production of CTXΦ, the CTX prophage does not yield virions because of the atypical arrangement of its prophage arrays (*4*).

Genetic hybrids between El Tor and classical biotypes of O1 *V. cholerae* were reported among sporadic isolates earlier in Bangladesh (*5*) and were named the Matlab variants after the place where they were first isolated. The Mozambique strains of *V. cholerae* likely evolved from an El Tor strain, which shed its CTX phage and acquired the classical prophage. Alternatively, strains like the Matlab variant may have spread to the African subcontinent. Whether introducing the CTX prophage in the El Tor genome background will increase pathogenicity, affect genomic stability, or enhance the epidemic-causing potential is uncertain. This subtle genetic change might also alter the effectiveness of current cholera vaccines which stimulate antitoxic as well as antibacterial immunity.
